# A machine learning approach for vocal fold segmentation and disorder classification based on ensemble method

**DOI:** 10.1038/s41598-024-64987-5

**Published:** 2024-06-23

**Authors:** S. M. Nuruzzaman Nobel, S. M. Masfequier Rahman Swapno, Md. Rajibul Islam, Mejdl Safran, Sultan Alfarhood, M. F. Mridha

**Affiliations:** 1 Department of Computer Science and Engineering, Bangladesh University of Business and Technology, Dhaka, 1216 Bangladesh; 2 Department of Electrical and Electronic Engineering, The Hong Kong Polytechnic University, Hong Kong, China; 3 Department of Computer Science, College of Computer and Information Sciences, King Saud University, P. O. Box 51178, 11543 Riyadh, Saudi Arabia; 4 Department of Computer Science, American International University-Bangladesh, Dhaka, 1229 Bangladesh

**Keywords:** Computational biology and bioinformatics, Machine learning

## Abstract

In the healthcare domain, the essential task is to understand and classify diseases affecting the vocal folds (VFs). The accurate identification of VF disease is the key issue in this domain. Integrating VF segmentation and disease classification into a single system is challenging but important for precise diagnostics. Our study addresses this challenge by combining VF illness categorization and VF segmentation into a single integrated system. We utilized two effective ensemble machine learning methods: ensemble EfficientNetV2L-LGBM and ensemble UNet-BiGRU. We utilized the EfficientNetV2L-LGBM model for classification, achieving a training accuracy of 98.88%, validation accuracy of 97.73%, and test accuracy of 97.88%. These exceptional outcomes highlight the system’s ability to classify different VF illnesses precisely. In addition, we utilized the UNet-BiGRU model for segmentation, which attained a training accuracy of 92.55%, a validation accuracy of 89.87%, and a significant test accuracy of 91.47%. In the segmentation task, we examined some methods to improve our ability to divide data into segments, resulting in a testing accuracy score of 91.99% and an Intersection over Union (IOU) of 87.46%. These measures demonstrate skill of the model in accurately defining and separating VF. Our system’s classification and segmentation results confirm its capacity to effectively identify and segment VF disorders, representing a significant advancement in enhancing diagnostic accuracy and healthcare in this specialized field. This study emphasizes the potential of machine learning to transform the medical field’s capacity to categorize VF and segment VF, providing clinicians with a vital instrument to mitigate the profound impact of the condition. Implementing this innovative approach is expected to enhance medical procedures and provide a sense of optimism to those globally affected by VF disease.

## Introduction

The vocal folds, referred to as the VF in the scientific literature, are dynamic anatomical structures inside the larynx^1–3^. The capacity for vocalization and sound production is contingent on the VF. Speech production involves the coordination of several anatomical structures, including the mouth and other articulators, after initiation of VF vibrations^4–7^. This process occurs when the air originating from the lungs passes through the larynx. VF diseases are significant because of their impact on communication^8^. Given the importance of VF in the communication process^9–11^, any disorder that affects them can hinder effective communication. This phenomenon could have a detrimental influence on interpersonal interactions and hinder individuals’ progress in their personal and professional lives. Furthermore, this phenomenon can potentially result in substantial social consequences. Effective communication^12–14^ is a fundamental aspect of human connection, and VF problems may provide challenges in maintaining this crucial element of daily life. Hence, it is essential to recognize, diagnose, and treat these disorders in order to enhance or reinforce an individual’s ability to communicate and engage with their surroundings. VF disorders include a collection of pathological conditions that affect the tissues comprising the VF^15,16^.

Common issues^17,18^ in the field of vocal health include VF polyps, which are benign lesions that arise from irritation or misuse of the VFs; laryngitis, an inflammation often triggered by infections or excessive vocal strain; VF paralysis, a condition characterized by impaired VF movement resulting from nerve damage; and Reinke’s oedema, a condition marked by the accumulation of fluid in the VF, often linked to smoking. VF segmentation from the larynx entails identifying and delineating the VF boundaries inside the laryngeal region using medical imaging or video analysis. Accurate segmentation plays a crucial role in diagnosing and treating VF abnormalities, as it provides^3,19,20^ medical workers with exact visual information, supports surgical planning, and advances current research on vocal disorders. VF segmentation^21,22^ is vital in speech therapy and rehabilitation as it enables therapists to effectively monitor patients’ advancements and make necessary adjustments to their treatment protocols. This research contributes to expanding our understanding of vocal physiology and pathology via biomechanical inquiries, shedding light on the intricate movements and functionalities of VF. Considering these factors, the discipline of voice and speech research and clinical practices have significant advantages over VF segmentation.

VF disorders present^23,24^ significant challenges for the medical community in contemporary times. It is crucial to promptly identify these issues; however, they can be overlooked. Limited therapy options may be available, necessitating the need for surgical intervention in some cases. There is a need to focus on preventive measures, such as promoting proper vocal hygiene. Accessing specialists^25–27^ for VF disorders, such as speech-language pathologists and otolaryngologists, might pose challenges in some regions. The potential consequences of this might negatively impact the provision of care. To address these difficulties, it is essential to focus on boosting knowledge, offering thorough education, and improving access to expert healthcare services as the main strategies.

### Motivation and contribution of this research

The motivation behind this research is rooted in the imperative need to enhance the precision and efficiency of diagnostic procedures concerning VF disorders, a significant yet often overlooked aspect of human health. VF disorder can have a significant impact on a people’s quality of life, affecting their communication abilities and, in many instances, their general state of well-being. Conventional diagnostic techniques require intrusive procedures or depend significantly on subjective evaluations, which may not consistently provide accurate or timely diagnoses of many illnesses. This study attempts to change the diagnostic process for VF diseases by utilizing modern ensemble methods in machine learning. The proposed approach integrates VF segmentation with disease categorization to create a unified system. This approach improves diagnostic accuracy and minimizes the time and resources required for assessment. This results in earlier and more accurate therapies, thereby enhancing the patient outcomes. Moreover, this system fills a significant void in existing medical technology, providing a flexible solution that can be customized for use in different healthcare environments worldwide. This research was motivated by a dedication to progressing medical technology and significantly enhancing diagnostic procedures for individuals suffering from VF disorders, contributing to improved health management and superior patient care.

**Figure 1:**
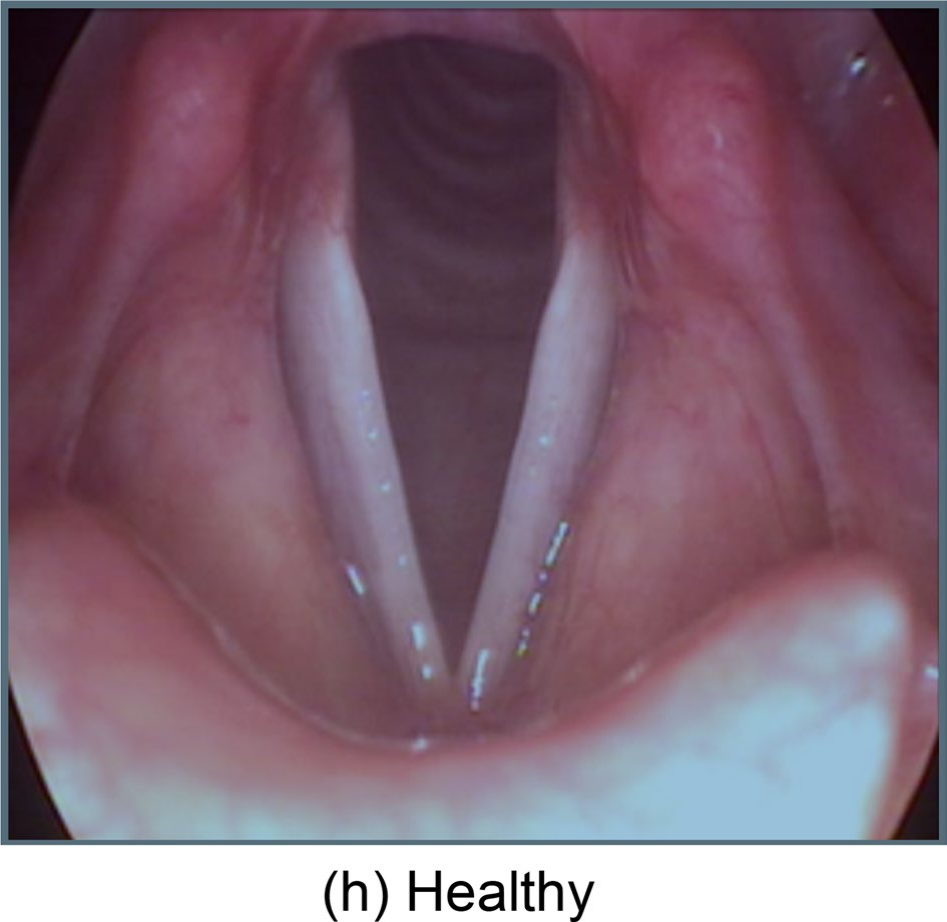
Here is a dataset image demonstrating healthy VFs and proper anatomical integrity.

**Figure 2:**
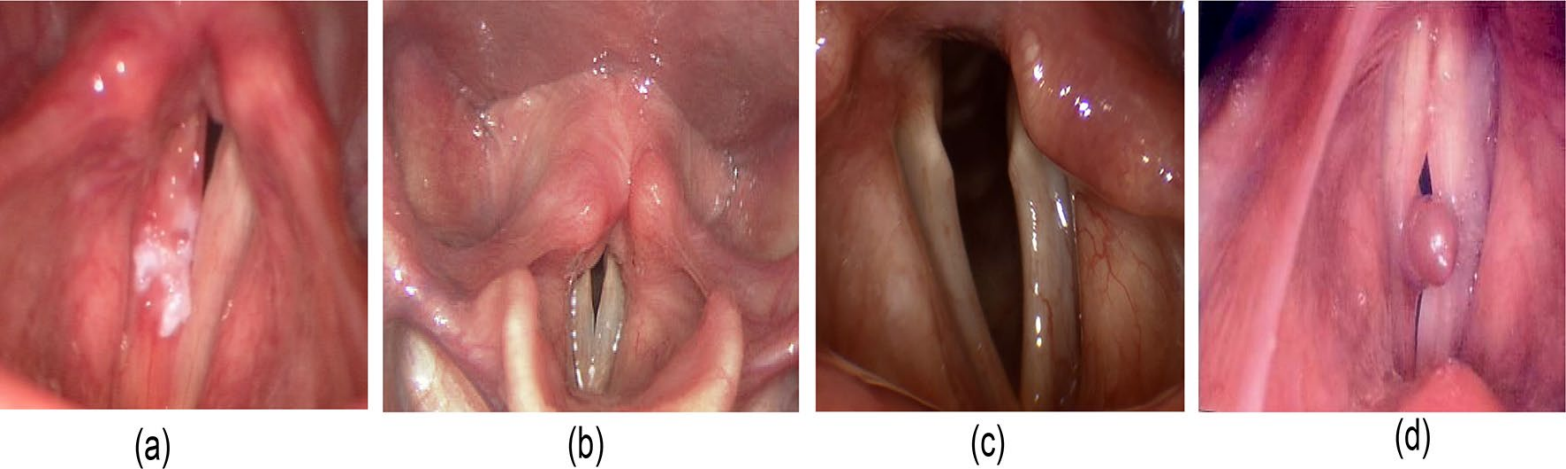
The following vocal fold diseases were found in our dataset: (**a**) carcinoma, (**b**) dysphonia, (**c**) paresis, and (**d**) polyp. These abnormalities impair the structure and function of the vocal folds.

In our inaugural deployment, we successfully developed pioneering methods for VF disease classification and segmentation. The method used was to build unique models for each task. We developed an ensemble EfficientNetV2L-LGBM model for VF disease classification and an ensemble UNet-BiGRU model for VF segmentation. We focused on the following five classes: dysphonia, polyp, paresis, carcinoma, and healthy. We categorize samples into four main groups: carcinoma, dysphonia, paresis, and polyp. A sample of classified healthy VF is shown in Fig. 1, and the characteristics of the four diseases are shown in Fig. 2.

This effective use of advanced models for the categorization and segmentation of diseases highlights our dedication to utilizing effective techniques to tackle significant obstacles in the field, opening the door to more precise diagnosis and focused therapies in the field of vocal pathology. The following are our responsibilities and contributions:

- Focusing on specific benchmark methods to ensure precise VF classification and segmentation for enhancing healthcare through improved automatic disease detection of medical images.
- Applying advanced machine learning and deep learning techniques for precise VF classification and segmentation in a single system.
- Developing an ensemble EfficientNetV2L-LGBM model for VF classification and UNet-BiGRU model for VF segmentation.
- Implementing fast, efficient, and accurate VF classification and segmentation in one system is useful for VF-affected patients.

### Organization of the paper

This study was divided into several sections, each serving a specific purpose. “Related work” provides an overview of the pertinent literature on the subject and forms the foundation for this inquiry. In “Research methodology” , the research plan is explained along with the technique used in this study. The results of the study are explained in “Result analysis” , where the analyses are presented. An in-depth discussion of the topic and critical evaluation of the results and implications are provided in “Discussion” . Finally, “Conclusion and future work” presents the study results and recommendations for the following research directions.

## Related work

Analyzing and identifying structures within medical images or data about the VF data is the primary goal of VF classification and segmentation. The aim is to promote research on speech and vocal problems, medical diagnosis, and treatment planning. Furthermore, by enhancing workflow efficiency in the healthcare industry and offering insightful information for medical education and training, these analyses aid in creating automated systems. Researchers are developing innovative approaches to VF categorization to increase diagnosis accuracy and provide critical new understandings of speech and voice problems. The various methods highlight a dedication to improving the VF analysis technology and medical knowledge.

Categorization of laryngeal illnesses according to VF morphology and vascular abnormalities, Turkmen et al. looked at a novel method for classifying VFs using machine learning and image processing, which made use of the edges of the VFs and visible blood vessels. They showed that shape characteristics and a specialized vessel centerline extraction process provided helpful information and that histograms of oriented gradients (HOG) descriptors recognized voice folds on video laryngostroboscopy images. Based on edge form and vascular structural parameters, they classified using a unique binary decision tree design. Sensitivities for polyps, nodules, laryngitis, sulcus vocalis, and healthy classes were 86%, 94%, 80%, 73%, and 76%, respectively, based on their examination of laryngeal images from 70 patients. According to the researchers, the results suggested that VF disorders may benefit from using visible arteries and VF form characteristics as prognostic indicators, improving diagnostic efficacy^28^. For the VF tumor classification, Gun et al. recommended deep deep-learning method to categorize tumor types into cysts, granulomas, leukoplakia, nodules, and polyps while concurrently detecting suspicious locations of benign VF tumors in laparoscopic images. Their method aimed to facilitate the early detection of benign tumors around the VF by a more straightforward self-prescreening at home. Twenty three laryngoscopic images were used to train, verify, and evaluate four convolutional neural network (CNN) models: Yolo V4, two Mask R-CNNs, and a single-shot detector. They found that Yolo V4 had the greatest F1-score for all tumor types (0.7664 for cysts, 0.9875 for granulomas, 0.8214 for leukoplakia, 0.8119 for nodules, and 0.8271 for polyps) among the four models they had used in their experiments. For every kind of tumor, Yolo V4 performed best for cysts and granulomas, whereas Mask R-CNN performed best for leukoplakia, nodules, and polyps. Their model with the lowest false-negative rate differed according to the tumor type. Furthermore, a nearly equal F1-score (0.8529) was shown by the embedded-operated Yolo V4 model compared to the computer-operated Yolo-4 model (188683)^29^.

By analyzing the voice acoustics of pediatric vocal nodule patients, Nagihan et al. examined 72 children with vocal nodules were examined. In images were captured from video recordings taken during the video stroboscopy test, and nodules were identified using the Image News program and categorized using the scale. Automatic segmentation was used to assess fundamental frequencies (mean F0), jitter (local%), shimmer (local%), and harmonicity (mean harmonics-to-noise [mean HNR]) in speech acoustic analysis using nodules/widths and VF as ratios. A significant negative correlation was found between the mean F0 value and nodule base/width ratio (*P* = 0.042, $r = -0.240$). Additionally, there was a statistically significant negative correlation between jitter (%) and vocal nodule base/width (*P* = 0.009, $r = -0.305$). Ultimately, a statistically significant positive correlation (*P* = 0.034, *r* = 0.324) was found between the mean HNR and width of the vocal nodules. The discriminant analysis correctly classified the degree of scale of the classifying variables at 73.6%^30^.

VF segmentation using a snake model, Allin et al. clips from a stroboscopic video showing the medial borders of the VF. Both components constitute the system. They first acquired a color transformation that met the fisher linear criteria to discern between the trachea and VF as well as possible. VF borders may coarsely segment the use of this change. Second, the identified contours were refined using an active contour formulation recently created for the Insight Toolkit. As an alternative to biassing the internal energy of the active contours for specific shapes, the system optimized the visual energy to draw attention to interest borders. Their study showed that by suppressing noisy artefacts that may confuse typical implementations, this modification of the image energy simplifies the contour extraction procedure. Their study examined phonation using stroboscopic video. The points on the perceived visual field borders were compared to those on the mechanically extracted contours. With a standard deviation of 3.6, the mean deviations for locations on the VFs minor axes were 2.2 pixels on average for all patients^31^. To classify VF leukoplakia, Zhenzhen et al. worked on image files in both white light and narrow-band imaging (NBI) that were generated and categorized into six classes: squamous cell carcinoma (SCC), mild dysplasia (MiD), moderate dysplasia (MoD), severe dysplasia (SD), and normal tissues (NT). Six traditional deep learning models were used to classify VF leukoplakia: AlexNet, VGG, Google Inception, ResNet, DenseNet, and Vision Transformer. The GoogLeNet, DenseNet-121, and ResNet-152 models were put into practice and showed outstanding classification performance. They achieved the most significant overall accuracy of 0.9583 for white light image classification and the highest overall accuracy of 0.9478 for NBI image classification. These three neural networks exhibit high sensitivity, specificity, and accuracy^32^.

Implementation of the classification System VF disease, Hertiana et al. presented research on categorizing VF diseases using digital image processing methods while working on digital image processing. The glottics contour on the VFs changed, exhibiting features suggestive of VF illness. Changes were divided into six categories: standard, paralysis, nodule, papilloma, cyst, and granuloma. The VF images were extracted before the classification procedure to extract the information or properties of the objects in the image. They extracted the VF glottis contour using form measurement and feature descriptions using the speeded-up robust features (SURF) technique, enabling analysis and classification. They require the vocal image to be in binary form to assess the glottis contour of the VF. The research used an approach based mainly on the active contour Chan-Vese algorithm to automatically acquire glottis region segmentation without human input to obtain binary image. The findings are shown as optimized glottis contour extraction, and 96.7% accuracy was attained in the classification training procedure using the K-Nearest Neighbor^33^. The application of computer-aided diagnostics proposed by Verikas et al. classified VF disorders, approaching this issue as a method of pattern identification. They used color, texture, and geometric shapes to create a clear and informative representation of VF images. The representation classified images into three categories: nodules, diffuse, and healthy, acting as a pattern classifier. Seven hundred eighty-five VF images obtained from the Department of Otolaryngology, Kaunas University of Medicine, Lithuania, were used to evaluate the proposed technique. When classifying a batch of unseen photos into three previously described classifications, an accurate classification rate of more than 87% was attained^34^. Categorized laryngeal diseases, Antanas et al. adopted Intelligent VF Image Analysis, using the following primary visual characteristics for analysis: color, shape, geometry, contrast, irregularity, and roughness. Researchers built a decision support system to automate the interpretation of VF images. This system uses several VF images to improve dependability and decrease variability between and among observers. Their approach used geometrical characteristics, color, and texture to extract significant information from images of the voice cords. They used a committee of artificial neural networks to classify images of the VF into nodular, diffuse, and healthy categories. After testing 785 photos of the VFs, the authors correctly classified nearly 93% of the images^35^.

By using dysphonic voices to distinguish between VF paralysis and vocal nodules, Valerio et al. used a machine learning strategy; it is essential to consider similarities in perception when treating VF paralysis (VFP) and vocal nodules (VN) in order to provide effective treatment. Using a dataset of speech recordings from 87 control patients, 85 VN-affected subjects, and 120 VCP-affected people, the authors developed a framework for identifying and distinguishing mental disorders. They used a gaussian support vector machine (GSVM) classifier and created their dataset within a tightly monitored clinical environment. They showed encouraging categorization findings, with accuracy levels of over 98% compared to those in good health. Furthermore, a remarkable 89.21% accuracy was obtained in distinguishing between VCP and VN. The results point to the possibility of automatically recognizing dysphonic voices and differentiating dysphonia aetiologias^36^. Recognizing VF injuries using Edges et al. created a model using clinically verified disease inference data. They take into account 13 instances that two medical professionals have verified. During the experiment, 1740 photos were captured from 13 movie cases. They employed a five-fold cross-validation technique for model training, validation, and testing. Randomly, 60% of the images (1044), 20% (348) for validation, and another 20% (348) for testing were selected. The EVC-DD model achieved 100% accuracy in identifying the three conditions necessary for the best possible experiment outcomes during the training phase. The averaged F1 score, averaged recall rate, averaged precision, accuracy, matthew’s correlation coefficient, and area under the curve for the EVC-DD model were 99.42%, 99.42%, 99.42%, 98.91%, and 99.57%, respectively. The EVC-DD model developed by the authors took approximately 400 seconds to train using 1740 photos. The EVC-DD model showed high concordance with clinical tests, and its training was time- and data-efficient, allowing for the rapid acquisition of new instances^37^.

The main problem is the difficulty in accurately classifying the disease and segmenting the VF. Furthermore, the performance of the current approaches can be improved. Scholars have continually been drawn to this field of study by the suggested ensemble EfficientNetV2L-LGBM and UNet-BiGRU models, which have generated great historical interest in resolving this difficulty.

## Research methodology

**Figure 3:**
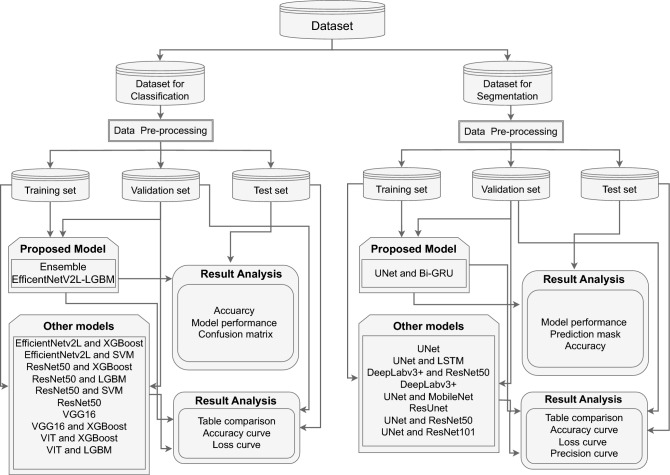
To achieve our research objectives, we have created a comprehensive workflow diagram that encapsulates our system architecture and processes, illustrating how the various components interact.

The method employs a sophisticated algorithm for classifying VF illnesses and segmenting VF. We developed a practical VF disease classification approach using an ensemble EfficientNetV2L-LGBM model. The ensemble UNet-BiGRU model was also used to accurately segment the VF. These two models, renowned for their velocity, can rapidly diagnose and segment VF disorders. The intricate arrangement of its constituents which harmoniously interact renders it unique. These components enhance the model’s capabilities, enabling it diagnose and segment disorders related to VF rapidly and precisely. This robust and all-encompassing model demonstrates our dedication for enhancing VF research and diagnostics. Figure 3 shows the detailed workflow of our system created for classifying VF diseases and segmenting folds. The procedure involves connecting datasets, performing model operations, classification and segmentation tasks, and displaying the results. Every phase is intricately integrated into the system design to guarantee efficient and precise analysis of VF conditions. Our system intends to establish a strong foundation for detecting and segmenting vocal fold illnesses, ultimately improving medical diagnostics and treatment planning.

### Dataset analysis and discussion

To develop our system, we used a large dataset available from Zenodo^38^, a robust repository housing an 804 megabyte (MB) image dataset. We utilized 24,000 (true and masked images) for the classification and segmentation. Out of the 24,000 (accurate and masked images) photos, 12,000 were utilized for classification purposes, while the remaining data consisted of masked images. This masked image serves the purpose of both the segmentation and representation of the original image. It is known that, classification cannot be performed using masked photos. Therefore, we selected 12,000 images specifically for classification, whereas the total number of images (including both masked and true images) was used for segmentation. To classify VFs, we have divided them into the following categories: the training dataset consisted of 10,377 photos, the validation dataset had 1152 images, and the testing dataset contained 1281 images. For segmentation, 18,000 samples were allocated to training, 3000 to validation, and 3000 for testing. Table 1 displays the quantity of data used for classification and segmentation.

**Table 1:** Distribution of data for VF classification and segmentation: this provides information on the total number of images as well as the counts that are assigned to training, test sets, and validation sets.

Domain	Training set	Validation set	Test set	Total data
Classification	10,377	1152	1281	12,810
Segmentation	18,000	3000	3000	24,000

This information is useful for developing and assessing models.

This dual-pronged approach facilitated intricate and comprehensive processing of the original dataset and meticulously extracted precise segmentation details. Furthermore, it allows for the adept utilization of segmented data in subsequent classification tasks, contributing significantly to the robustness and accuracy of our system’s outcomes.

### Data preprocessing

We effectively resolved the imbalance problem in our dataset by employing a range of preprocessing approaches designed explicitly for the classification and segmentation tasks. Regarding image resizing, we established a consistent size of $256\times 256\times 3$ pixels to provide uniformity throughout our dataset. In the data classification and segmentation domain, our primary focus was on the labels assigned to the images. To streamline the numerical analysis, we used label encoding, which efficiently transforms categorical variables into a numerical format. After performing the encoding stage, we improved the resilience of our data by normalizing it, which is an essential operation for classification and segmentation tasks. This rigorous preparation procedure guarantees uniformity in image the dimensions and enhances the dataset for later classification and segmentation analyses. Another technique is standardization, where *z* represents the standardization score, *x* is the data value, $\mu$ represents the mean, and $\sigma$ represents the standard deviation. The formula is shown in Eq. (1).

1 $$\begin{aligned} z=\frac{(x-\mu )}{\sigma } \end{aligned}$$

We adopted a practical technique by leveraging arrays, where we meticulously divided array elements to systematically enhance our data quality. By processing each element individually, we significantly improved the overall quality of the data. In addition, we employed the “To Categorical Label” methodology, which is a crucial stage in our procedure. Using this strategy, we can easily convert non-numeric categorical labels into a format that works with our models. This conversion involved encoding these labels into numerical representations to ensure our models could easily comprehend and operate using the data, thereby improving their accuracy and performance.

### Execution of ensemble EfficientNetV2L-LGBM model to classify VF disease

The EfficientNetV2L model, which is our feature extraction architecture, was carefully created to maximize neural execution. It begins by processing the input at $256\times 256$ dimensions using a series of layers. We combined many elements inside each layer to improve the effectiveness of the neuron execution. In particular, we have paid special attention to using MB-Conv and fused MB-Conv approaches. The performance of our model’s was optimized by configuring the neurons with a $3\times 3$ kernel. Within each layer, these finely defined neurons strive to predict a more precise output, ultimately resulting in a $1\times 1$ convolution. The system then uses an LGBM model classifier capable of categorizing five different illnesses using the characteristics of this complex neural network.

#### EfficientNetV2L model executing process

**Figure 4:**
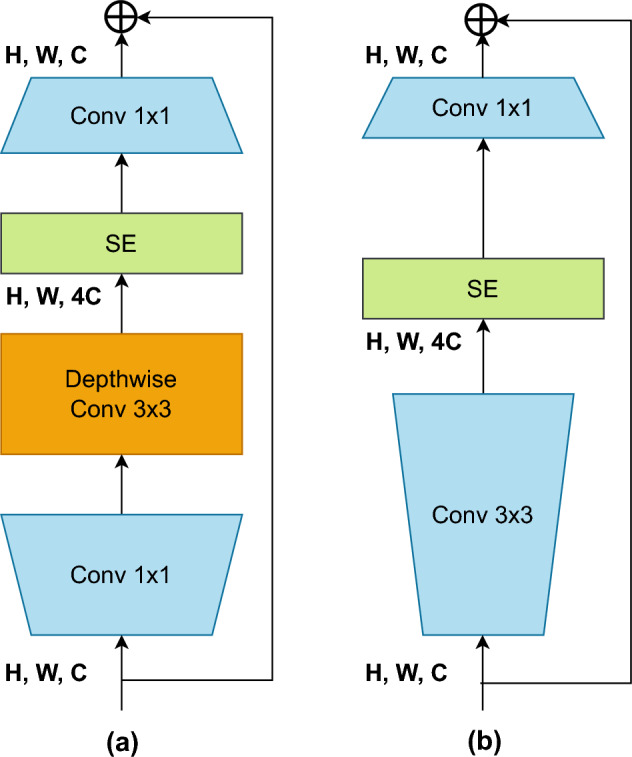
The EfficientNetV2L model’s architectural components, which highlight the MBConv (mobile inverted bottleneck convolution) and fused convolution layers, are essential components that enhance the model’s effectiveness and performance in deep learning tasks.

**Figure 5:**
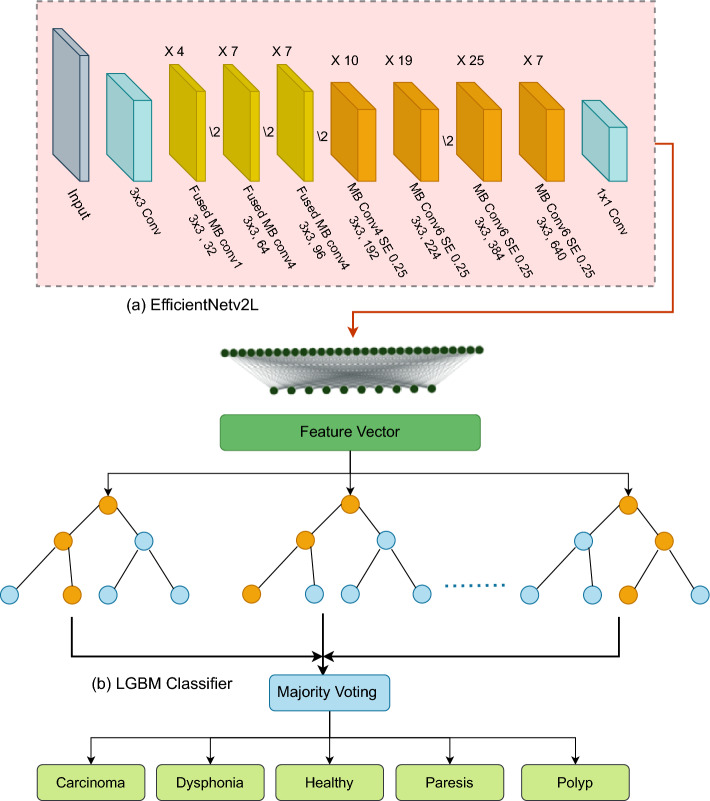
The proposed EfficientNetV2L-LGBM model for vocal fold disease classification presents a comprehensive architecture that showcases the integration of EfficientNetV2L’s convolutional backbone with LightGBM. This innovative fusion leads to improved performance in accurately categorizing vocal fold disorders.

EfficientNetV2L’s large depthwise convolutions provide another training barrier. Although depthwise convolutions generally cannot fully use current accelerators, they contain fewer parameters and FLOPs than regular convolutions. Recently, Fused-MBConv was employed to use servers or mobile accelerators. As seen in Fig. 4a, it substitutes a single normal conv $3\times 3$ for the depthwise conv $3\times 3$ and expansion conv $1\times 1$ in MB Conv. We progressively replaced the old MBConv in EfficientNetV2L with Fused-MBConv to methodically compare the two building blocks. Replacing all blocks with Fused-MBConv dramatically increased the parameters and FLOPs while slowing down training. However, when the model execution starts, Fused-MBConv can enhance the training time with minimal overhead on parameters and FLOPs. This method is shown in Fig. 4b. Determining the ideal combination of these two building blocks, MBConv and Fused-MBConv, is difficult. We used a neural architecture search to automatically find the optimal combination. Figure 5a illustrates the execution procedure of the EfficientNetV2L model.

While examining operators, we investigate MBConv and Fused MBConv, each providing a unique combination of kernel size and stride. This presentation thoroughly explains the channels and layers responsible for channel execution. It also includes a detailed description of all levels involved. Table 2 presents a comprehensive overview of this information, including a thorough analysis of operators, strides, channels, and related layers.

**Table 2:** An explanation of the MBConv and Fused-MBConv blocks in the EfficientNetV2L architecture, including important settings and parameters inside these vital parts.

Stage	Operator	Stride	Channels	Layer
0	Conv,3$\times$3	2	32	1
1	Fused-MBConv1,3$\times$3	1	32	49
2	Fused-MBConv4,3x3	2	64	7
3	Fused-MBConv4,3$\times$3	2	96	7
4	MBConv4 SE 0.25,3$\times$3	2	192	10
5	MBConv6 SE 0.25,3$\times$3	1	224	19
6	MBConv6 SE 0.25,3$\times$3	2	384	25
7	MBConv6 SE 0.25,3$\times$3	1	640	7
8	Conv1x1 & Pooling & FC	–	1280	1

The design of the EfficientNetV2L model is highly intricate and consists of several parts, including resolution scaling, depth, and breadth. The mathematical formula for achieving accurate and efficient neural network topologies involves scaling these parameters.

Compound scaling is the foundation for EfficientNetV2L execution, which balances the number of layers, channels, and image resolution in depth and breadth. It is represented by:

$$\begin{aligned} EfficientNetV2L\left( B^{depth}_d\,\, B^{width}_w \,\, B^{resolution}_r\right) \end{aligned}$$

where, $B^{depth}_d$ is the depth scaling factor, $B^{width}_w$ is the width scaling factor, $B^{resolution}_r$ is the resolution scaling factor. Empirical investigations are typically used to estimate the scaling factors $B^{depth}_d\,\, B^{width}_w \,\, B^{resolution}_r$ to balance model accuracy and size. The algorithm used to compute these variables often seeks to optimize the trade-off between these variables.

EfficientNetV2L design presents a compound scaling technique that uses a compound coefficient to scale all dimensions of depth, breadth, and resolution equally. Defining, compound coefficient: denoted by $\phi$, depth: number of layers *(d)*, width: width multiplier for convolutional layers *(w)*, and resolution: input image resolution *(r)*. The subsequent execution was as follows:

2 $$\begin{aligned} d= & {} \alpha ^{\phi } \end{aligned}$$

3 $$\begin{aligned} w= & {} \beta ^{\phi } \end{aligned}$$

4 $$\begin{aligned} r= & {} \gamma ^{\phi } \end{aligned}$$

Here, the constants $\alpha , \beta , \gamma$ has been determined by empirical means. The compound coefficient $\phi$ is responsible for concurrently controlling depth, width, and resolution scaling.

#### LGBM model executing process

The LGBM framework is commonly used to implement the gradient boosting decision tree (GBDT) model in machine learning. The study employed LGBM, an efficient data categorization technique used in various fields, including industry, health, and economics, to address the belt conveyor malfunction diagnostic problem. The exclusive feature bundle (EFB), gradient-based one-side sampling (GOSS), and histogram algorithms were combined in the LGBM model based on the GBDT learning model. This may ensure model correctness while enhancing the learning efficiency of the model. Figure 5b illustrates the execution procedure of the LGBM model.

Initially, given the presence of a training set $X=x_{1},x_{2},...,x_{n}$, the LGBM model starts by initializing a tree with a constant value:

5 $$\begin{aligned} y^{(0)}_{i}=f_{0}=0 \end{aligned}$$

Let $y^{(0)}_{i}$ represent the forecast of the $i_{th}$ case at iteration *t*.

Next, the subsequent tree is trained using the lowest loss function.

6 $$\begin{aligned} f_{t}\left( x_{i} \right) = arg\,min\, L_{t}=arg\,min\,L\left( y_{t},y_{i}^{(t-1)}+f_{t}\left( x_{i} \right) \right) \end{aligned}$$

Let $f_{t}\left( x_{i} \right)$ denote the learning model of the $t_{th}$ decision tree. The subsequent model is projected as:

7 $$\begin{aligned} y^{t}_{i}=y_{i}^{(t-1)}+f(x_{i}) \end{aligned}$$

Equations (6) and (7) were iterated until the model satisfies the termination condition. The ultimate formula for the model is:

8 $$\begin{aligned} y_{i}=\sum _{t=0}^{Z-1}f_{t}\left( x_{i} \right) \end{aligned}$$

Let *Z* represent the total number of iterations.

#### Optimization process of ensemble EfficientNetV2L-LGBM model

Usually, backpropagation techniques are used to train machine learning model $K_{s}$ end-to-end while reducing the cross-entropy loss:

9 $$\begin{aligned} E=-\frac{1}{n}\sum _{i=1}^{n}\sum _{k=1}^{n}\,\,1\left( y_{ik}=k \right) ln\left( \frac{exp\left( \left( w^{out}_k \right) ^T \right) }{\sum _{j=1}^{C}exp\left( \left( w^{out}_j \right) ^T \right) h^{out}_j} \right) . \end{aligned}$$

where *C* is the number of classes, $y_{ik}$ is the prediction probability for sample *i* and class *K*, *n* is the number of training samples, $h_{k}^{out}$ is the output of the final hidden layer, and $w_{k}^{out}$ is the weight matrix from that hidden layer to the output layer. Equation (9) is an indication function that returns 0 otherwise and 1 if the assertion is true.

We must jointly minimize the two cross-entropy errors, $E_{1}$ and $E_{2}$, for our proposed EfficientNetV2L, where we have two outputs. To do this, we maximize the weighted sum of the two errors:

10 $$\begin{aligned} E=\gamma _{1}E_{1}+\gamma _{2}E_{2} \end{aligned}$$

where the output trade-off of the two branches is controlled by two positive hyper-parameters, $\gamma _{1}$ and $\gamma _{2}.$

11 $$\begin{aligned} E=\gamma _{1}\left( E_{1}+\frac{\gamma _{2}}{\gamma _{1}}E_{2}\right) \end{aligned}$$

Because scaling the loss *E* by a positive parameter $\gamma _{1}$ does not affect the minimization issue, this equation requires only one parameter, $\lambda =\frac{y_{2}}{y_{1}}$. As there should not be a preference for one output over another, we set $\lambda =1$ for this process.

The provided description thoroughly elucidates the operational approach for a classification problem utilizing the EfficientNetV2L-LGBM model. This study provides a methodical examination of the model’s operation, with a comprehensive explanation of each stage. By following this technique, we can construct our classification task model.

#### Hyperparameter tuning for EfficientNetV2L-LGBM model

We have incorporated hyperparameter tuning into our EfficientNetV2L-LGBM model, which was used for performing classification tasks. Hyperparameter tuning is essential in machine learning and deep learning, because it enables the maximization of model performance and generalization capacity. By manipulating hyperparameters such as the learning rate, the number of hidden layers, or regularization strength, we can optimize models to achieve higher accuracy and mitigate problems such as overfitting or underfitting. This method is crucial for enhancing the resilience and efficacy of machine learning algorithms, ultimately resulting in more dependable and efficient models across diverse applications. We utilized a tailored methodology to accomplish a classification assignment that merged the EfficientNetV2L deep learning model with the LGBM machine learning model. We implemented an approach that involved thorough parameter adjustments for both types of models. Our hyperparameter tuning for the deep learning model (EfficientNetV2L) was to optimize the kernel size, padding, and pooling parameters (serial numbers: 1 to 3). Concurrently, we performed parameter tuning for the LGBM machine learning model, explicitly focusing on Serial numbers 4 to 11. The adjustments included modifying the number of layers, maximum tree depth (max_depth), learning rate, number of estimators (n_estimators), objective function, minimum child weight (min_child_weight), minimum split gain (min_split_gain), and random seeds (random_state). We systematically tested and optimized each parameter for optimal performance and generalization in our classification challenge. Table 3 displays the comprehensive results of implementing hyperparameter tuning for the EfficientNetV2L-LGBM model. This highlights the individual settings of the parameters and their respective effects on the model’s performance metrics. This comprehensive strategy combines the advantages of deep learning and machine learning approaches to optimize classification accuracy and resilience.

**Table 3:** Hyperparameter tuning experiment for classification task model.

Serial number	Parameter	Search space	Selected value
1	Kernel	[$3 \times 3, 5 \times 5$]	$3 \times 3$
2	Padding	[Same, valid]	Same
3	Pool	[Max]	Max
4	Num_layer	[3, 31, 42]	3
5	Max_depth	[7, 11, 5, 3]	7
6	n_estimators	[12, 13]	12
7	Learning rate	[0.1, 0.2, 0.3]	0.2
8	Objective	[Multiclass]	Multiclass
9	Min_child_weight	[0.0001, 0.0002]	0.0001
10	Min_split_gain	[0.1, 0.2, 0.3]	0.3
11	Random_state	[42, 69]	42

#### Novelty of EfficientNetV2L-LGBM model for VF classification

The main technical innovation of the image classification pipeline is its unique combination of machine learning and gradient boosting methods. Initially, using a pre-trained EfficientNetV2L CNN model for feature extraction enabled the efficient collection of hierarchical visual representations from input images, acquiring effective and discriminative features. Subsequently efficient classification can be achieved by compressing the retrieved characteristics and utilizing LightGBM, a machine-learning framework that boosts gradients. This combination exploits the advantages of both approaches: the deep learning model captures complex visual patterns, whereas the boosting technique improves the interpretability and generalization. The smooth incorporation of these approaches provides a potent and adaptable solution for tasks involving image classification, ensuring enhanced performance and resilience in the VF domain and datasets.

Additionally, the EfficientNetV2L-LGBM model presents a novel integration of convolutional neural networks (CNNs) and gradient-boosting machines Specifically designed for categorizing VF disorders. This ensemble model combines the advantages of EfficientNetV2L, a cutting-edge CNN architecture recognized for its remarkable efficiency and scalability, with LGBM, a robust and high-performance gradient boosting framework that is particularly adept at handling tabular data. The uniqueness of this approach is found in its two-step processing, where EfficientNetV2L initially extracts profound and intricate characteristics from VF imagery, guaranteeing a thorough portrayal of fundamental clinical states. Subsequently, these characteristics are inputted into the LGBM model, which carries out the categorization process. This ensemble methodology improves the ability of the model to detect subtle patterns and anomalies in the data that could indicate different VF disorders, resulting in a considerable improvement in classification accuracy. Moreover, integrating a CNN with a gradient boosting method enhances the efficiency of the model in several evaluation metrics, such as precision and recall, by efficiently balancing the compromises between bias and variance. This novel methodology enhances the precision of diagnosis and guarantees the strength and applicability across a wide range of datasets. The EfficientNetV2L-LGBM model is a significant breakthrough in machine learning applications in the medical domain, providing a more accurate, dependable, and efficient tool for identifying VF disorders.

###  Execution of ensemble UNet-BiGRU model to segment vocal fold

We used a UNet architecture in our VF segmentation method, with a kernel of three and a $256 \times 256$ input size. To improve the accuracy of VF segmentation, we modified the model to include BiGRU (Bidirectional Gated Recurrent Unit). Because of its complex layering and sophisticated processing skills, BiGRU has significantly enhanced segmentation accuracy. This feature has substantially contributed to attaining more precise and refined segmentation outcomes for VF images.

#### UNet model executing process

**Figure 6:**
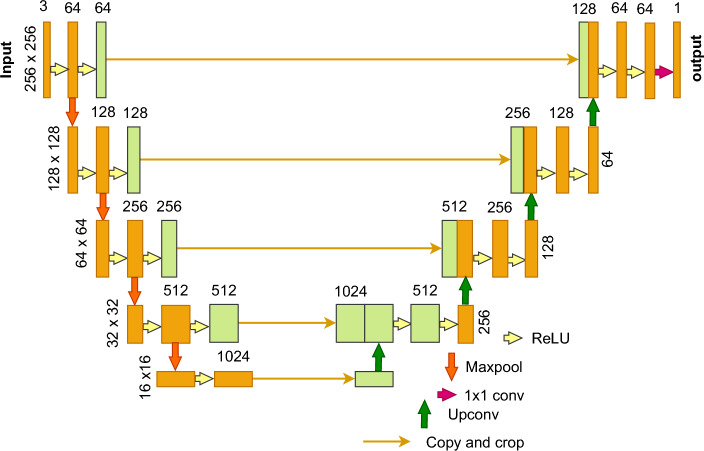
Representing the UNet architecture, exhibiting its characteristic shrinking and wide paths intended for semantic division applications in computer vision and image processing.

UNet is a well-liked network for semantic segmentation tasks in medical image processing, and is frequently utilized as a baseline^39^. The two components of UNet architecture are upsampling and feature extraction. An encoder-decoder structure is the term most often used to describe this arrangement. The network gets its name because it looks as the letter *U*. The input image is first pooled and convolved, as seen in Fig. 6. The image was pooled four times in the original UNet study, yielding features with sizes of $128\times 128, 64\times 64, 32\times 32, 16\times 16$. To retain the channel information, the $16 \times 16$ feature map is upsampled to create a $32 \times 32$ feature map, concatenating with the $32 \times 32$ feature map that came before it. Subsequently, the concatenated feature map undergoes convolution and upsampling, yielding a $64 \times 64$ feature map that is concatenated with the preceding $64 \times 64$ feature map. A $256\times 256$ prediction result, which is the same size as the input image can be achieved after four rounds of upsampling. Stochastic gradient descent is a method used to train the UNet. The input image with a constant border width is larger than the output image owing to unpadded convolution. In this case, the significant batch input satellite image reduces the overhead problem, and GPU usage enhancement reduces the batch to a single image. Owing to the increased training sample size and updating of the current optimization process, a significant momentum value is used.

12 $$\begin{aligned} g_{e}\left( y \right) =exp\left( r_{e}\left( y \right) \right) /\left( \sum _{e=1}^{E} exp\left( \acute{r}_{e}\left( y \right) \right) \right) \end{aligned}$$

This equation represents the softmax function, which applies the activation function to the input $r_{e}\left( y \right)$, considering the number of feature channels at the pixel locations $y\,\epsilon \,\Omega$ and $\Omega \subset z^{2}$. Here, the function $g_{e}\left( y \right)$ represents an approximation with the class denoted as *’E’*. The result of the approximation function is 1 when the maximal value $r_{e}\left( y \right)$ is reached and 0 for all other circumstances. The cross function encompasses all variations in location $g_{be}\left( y \right)$, starting from 1, and is mathematically represented as:

13 $$\begin{aligned} K=\sum _{y\epsilon \Omega }^{}w\left( y \right) log(\,g_{be}\left( y \right) ) \end{aligned}$$

The weight map, denoted as $\Omega \rightarrow R$, is used to assign value to certain pixels, whereas the real label of a pixel is referred to as $\Omega \rightarrow \left\{ 1,...., E \right\}$. In this approach, the weight map is calculated for ground truth segmentation to address the variation in pixel frequencies within the training dataset. This allows the network to effectively learn the boundaries of minor separations. Therefore, the weight map is represented as:

14 $$\begin{aligned} w\left( y \right) =w_{c}\left( y \right) +w_{0}\,exp\left( \frac{-\left( a_{1}\left( y \right) +a_{2}\left( y \right) \right) }{2\gamma ^{2}}^{2} \right) \end{aligned}$$

The term $a_{2}:\Omega \rightarrow R$ represents the distance between the second closest cell and border. $a_{1}:\Omega \rightarrow R$ represents the distance to the nearest cell border. The weight map used is called $w_{c}:\Omega \rightarrow R$, and which helps preserve of the frequencies of different classes.

In the network training process, the model’s parameters were initialized randomly, and the training set was used as an input to train the model. The loss of the model was determined by calculating the average cross-entropy loss using the following loss function:

15 $$\begin{aligned} loss=-\frac{1}{m}\sum _{i=1}^{m}\left[ x_{i}\,log\left( z_{i} \right) + (1-x_{i})\, log (1-x_{i})\right] \end{aligned}$$

In this context, the variable m denotes the size of the mini-batch, whereas $x_{i}$ and $z_{i}$ refer to the predicted and true values of the $i_{th}$ sample within each batch.

The optimized version of UNet utilizes the initial two layers to gather low-level characteristics, whereas the final three layers extract high-level characteristics.

16 $$\begin{aligned} L_{e}=\sum _{i=1}^{x}\sum _{j=1}^{y}\left[ G_{e}\,log\left( s_{e} \right) + \left( 1-G_{e} \right) log\,\left( 1-s_{e} \right) \right] \end{aligned}$$

The pixel locations (*I*, *j*) correspond to the anticipated edge map while represent the true map. The width and height of the feature map are denoted as *x* and *y*, respectively. These values were used to extract segmented portions of the pupil.

The UNet provides several benefits. First, the field of view of the feature map expands as the network layer gets deeper. Deep and profound features are valueable because shallow convolution concentrates on textural features, and deep networks concentrate on crucial aspects. Second, more information is frequently missing from the feature map edges of more significant sizes produced by deconvolution. This is a result of certain edge features being lost during downsampling and irretrievable during upsampling. Thus, an edge feature can be obtained via feature splicing.

#### BiGRU model executing process

**Figure 7:**
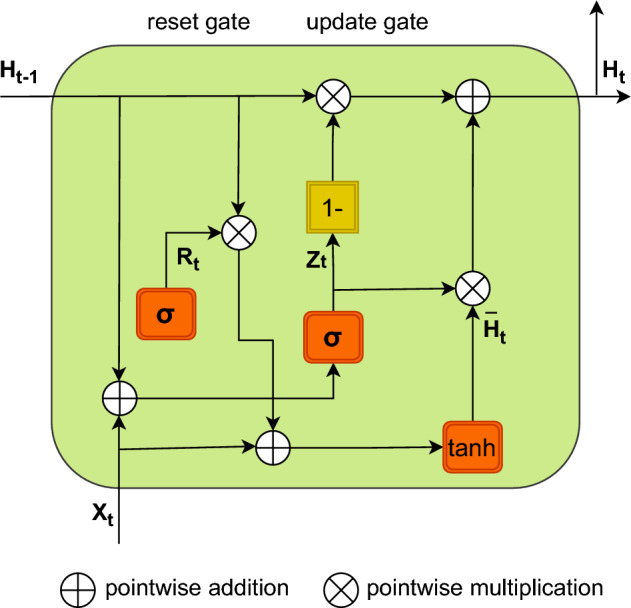
Visualization illustrates the basic GRU architecture, which is essential for simulating sequential data in machine learning tasks, demonstrating the internal workings and information flow of a recurrent neural network.

The gated recurrent unit (GRU) is a streamlined variant of the long short-term memory (LSTM) neural network, both of which belong to the family of recurrent neural networks (RNNs). However, the LSTM and GRU integrate the input and forget gates into a single update gate. The fundamental framework is illustrated in Fig. 7.

We assume that the number of hidden units is denoted as *h*. The small-batch input at a certain time step *t* may be represented as $x_{t}\epsilon \,R^{n*d}$, whereas the hidden state at the previous time step $t-1$ is denoted as $h_{t-1}\epsilon \,R^{n*h}$. The output hidden state *h* of a single GRU at the current time step *t* can be expressed as:

17 $$\begin{aligned} R_{t}= & {} \sigma \left( X_{t}W_{xr}+H_{t-1}W_{hr}+b_{r} \right) \end{aligned}$$

18 $$\begin{aligned} Z_{t}= & {} \sigma \left( X_{t}W_{xz}+H_{t-1}W_{hz}+b_{z} \right) \end{aligned}$$

19 $$\begin{aligned} \tilde{H_{t}}= & {} tan\,h\left( X_{t}W_{xh}+\left( R_{t} \odot H_{t-1} \right) W_{hh}+b_{h} \right) \end{aligned}$$

20 $$\begin{aligned} H_{t}= & {} \left( 1-Z_{t} \right) \,\odot H_{t-1}+Z_{t}\odot \,\tilde{H_{t}} \end{aligned}$$

The sigmoid activation function, denoted as $\sigma$, $\sigma \left( x \right) =1/1+e^{-x};W_{xr},W_{hr},W_{xz},W_{hz}$ represents the weights linking the input layer and reset gate, hidden layer and reset gate, input layer and update gate, and hidden layer and update gate, respectively. The terms $b_{r}$ and $b_{z}$ refer to the bias values of the reset gate and update gate’s, respectively. ${H_{t}}$ refers to the concealed condition at the present moment. Step $t;\odot$ denotes the process of multiplying two matrices. *Tanh* is a hyperbolic tangent activation function defined by the following formula:

21 $$\begin{aligned} tanh\left( X \right) =1-\frac{2}{1+e^{-2x}} \end{aligned}$$

**Figure 8:**
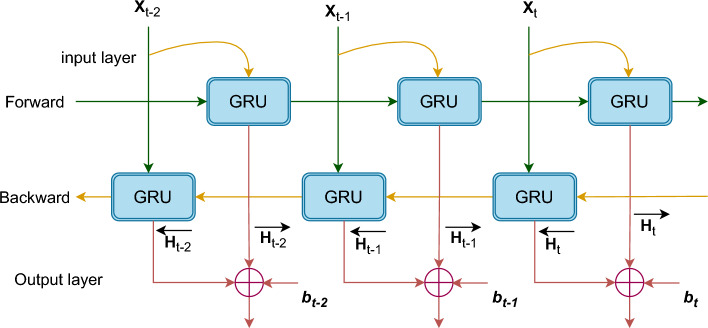
Diagram of Bidirectional GRU (BiGRU) architecture: this diagram shows two GRU layers that work together to capture sequential data in both directions. This allows for a more thorough comprehension of temporal dependencies in both phases.

The execution process of the BiGRU model, includes the input, forward, backward, and output layers. Nevertheless, the GRU architecture is unidirectional; hence, this study employed BiGRU, as shown in Fig. 8.

BiGRU is a neural network that consists of both forward-propagating and backward-propagating GRU units. The hidden layer state $H_{t}$ of the BiGRU is determined by the current input $X_{t}$, the output $\overrightarrow{H_{t}}$ of the forward hidden layer, and the output $\overleftarrow{H_{t}}$ of the backward hidden layer at time step *t-1*.

22 $$\begin{aligned} \overrightarrow{H_{t}}= & {} GRU\left( X_{t},\overrightarrow{H}_{t-1} \right) \end{aligned}$$

23 $$\begin{aligned} \overleftarrow{H_{t}}= & {} GRU\left( X_{t},\overleftarrow{H}_{t-1} \right) \end{aligned}$$

24 $$\begin{aligned} {H_{t}}= & {} w_{t}\overrightarrow{H}_{t}+v_{t}\overleftarrow{H}_{t}+b_{t} \end{aligned}$$

The input vector is encoded into the GRU hidden state, represented by $w_{t}$ and $v_{t}.$ These states correspond to the weights of the forward hidden layer $\overrightarrow{H_{t}}$ and the backward hidden layer $\overleftarrow{H_{t}}$ of the BiGRU at time *t*. Additionally, $b_{t}$ represents the bias of the hidden layer state at time *t*.

**Figure 9:**
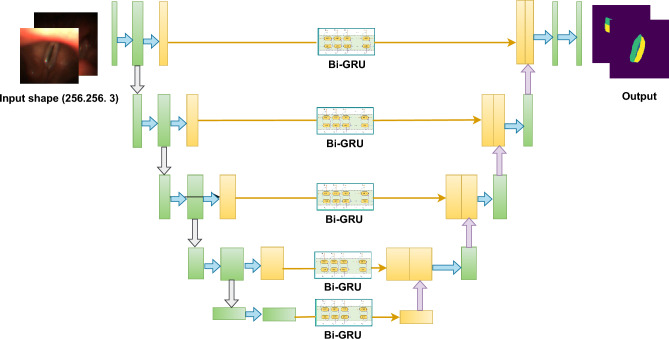
Schematic representation of a custom UNet-BiGRU model architecture designed for precise VF segmentation. The model integrates UNet’s encoding–decoding structure with bidirectional gated recurrent units (BiGRU) to enhance its ability to segment VF regions accurately.

The accompanying description comprehensively explains the operational methodology for a segmentation task using the UNet-BiGRU model. This execution offers a systematic analysis of the functioning of the UNet-BiGRU model, presenting a detailed description of each phase. We can create our segmentation task model by adhering to this procedure. Figure 9 shows the processing architecture of the ensemble UNet-BiGRU model.

#### Hyperparameter tuning for UNet-BiGRU model

We conducted a hyperparameter tuning experiment for the UNet-BiGRU model used in the segmentation task. We tested various parameters for this ensemble machine learning model, including decay, optimizer, learning rate, dropout, batch size, stride, and padding. This experiment aimed to assess the effectiveness of different parameters on our model. Table 4 shows the hyperparameter tuning experiment for the UNet-BiGRU model.

**Table 4:** Hyperparameter tuning experiment for segmentation task model.

Parameter	Search space	Selected value
Decay	[0.01, 0.0001, 0.00002]	0.00002
Optimizer	[Adam, RMSprop, Nadam]	RMSprop
Learning rate	[1e−4, 1e−4, 2e−4, 2e−3]	2e−4
Dropout	[0.1, 0.2]	0.2
Batch size	[8, 16, 32, 64]	16
Stride	[$1\times 1, 2\times 2$]	$1\times 1$
Padding	[Same, valid]	Same

#### Novelty of UNet-BiGRU model for VF segmentation

The main technical innovation of the proposed model is the integration of the UNet and BiGRU architectures for image segmentation. The UNet architecture effectively captures spatial information using its encoder–decoder structure, whereas the BiGRU model collects bidirectional temporal connections. By integrating various designs, the model successfully merges spatial and temporal information, leading to improved segmentation precision. The model can achieve more accurate segmentation results by combining the spatial awareness of UNet and temporal context of BiGRU. This approach makes it especially useful for tasks that require both spatial and temporal features to be considered, such as medical image analysis, video processing, and autonomous systems.

The novelty of the UNet-BiGRU model lies in its ability to process and integrate temporal dependencies across images, a feature largely absent in traditional convolutional neural networks used for segmentation tasks. This integration enhances the ability of the model to make consistent and accurate predictions when segmenting sequential data. As a result, a more thorough and dependable examination of the movement and structure of the VF is required. The combination of the spatial depth of UNet and the sequential depth of BiGRUs in our model is expected to establish a new standard for medical image segmentation, especially in situations where comprehending dynamic biological structures is essential. This method improves the capacity of the model to make correct predictions and makes it more useful in clinical contexts, where precise and rapid diagnosis is crucial.

## Result analysis

This section presents the results of our VF classification and segmentation. Our classification model’s integration of ensemble EfficientNetV2L and LGBM achieved outstanding performance. In addition, our segmentation model, which combined ensemble UNet and BiGRU, demonstrates remarkable results that are strongly influenced by their performances.

### Ensemble EfficientNetV2L-LGBM as classification model results

The ensemble model, which combines EfficientNetV2L and LGBM, accurately classifies four VF diseases: dysphonia, polyps, paresis, and carcinoma. Here, we provide a thorough summary of the VF illness categorization outcomes.

#### Accuracy and loss of vocal fold disease classification

To assess the accuracy of our working model for VF illness classification, we evaluated it using several key metrics: True Positive (TP), True Negative (TN), False Positive (FP), and False Negative (FN). This matrix shows cases incorrectly predicted as positive, with False Negative (FN) representing instances incorrectly predicted as negative. These measures are essential for evaluating the performance of a machine learning model, and helping in precision, recall, and overall accuracy assessments. Together, these numbers reveal the accuracy of the model in classifying the VFs.

25 $$\begin{aligned} Accuracy=\,\frac{TP+TN}{TP+TN+FP+FN} \end{aligned}$$

**Figure 10:**
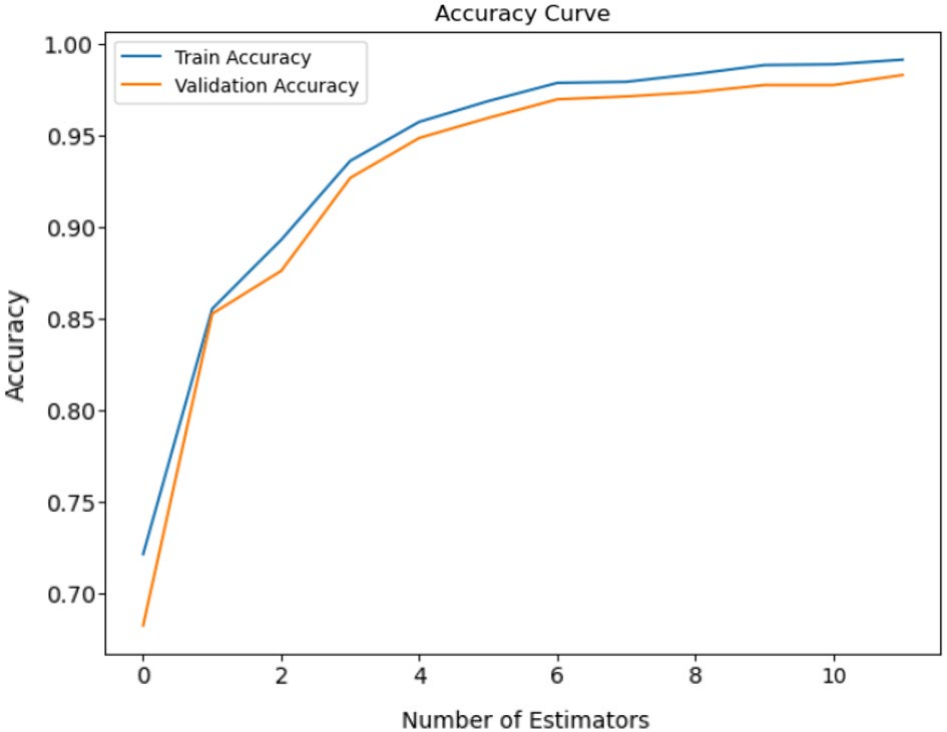
Our proposed model, the EfficientNetV2L-LGBM classifier, has a training and validation accuracy curve that shows the model’s convergence and generalization abilities by showing learning trends and the relative performance of training and validation datasets.

We successfully achieved a remarkable training accuracy of 98.88% for classifying the VFs. In addition, the validation accuracy was 97.73%. The results emphasize the high level of accuracy attained in the classification training and demonstrate the usefulness of our validation method. The accuracy of training and the effectiveness of validation enhanc the strength of our categorization system. Figure 10 presents the training and validation accuracy with number of epochs.

**Figure 11:**
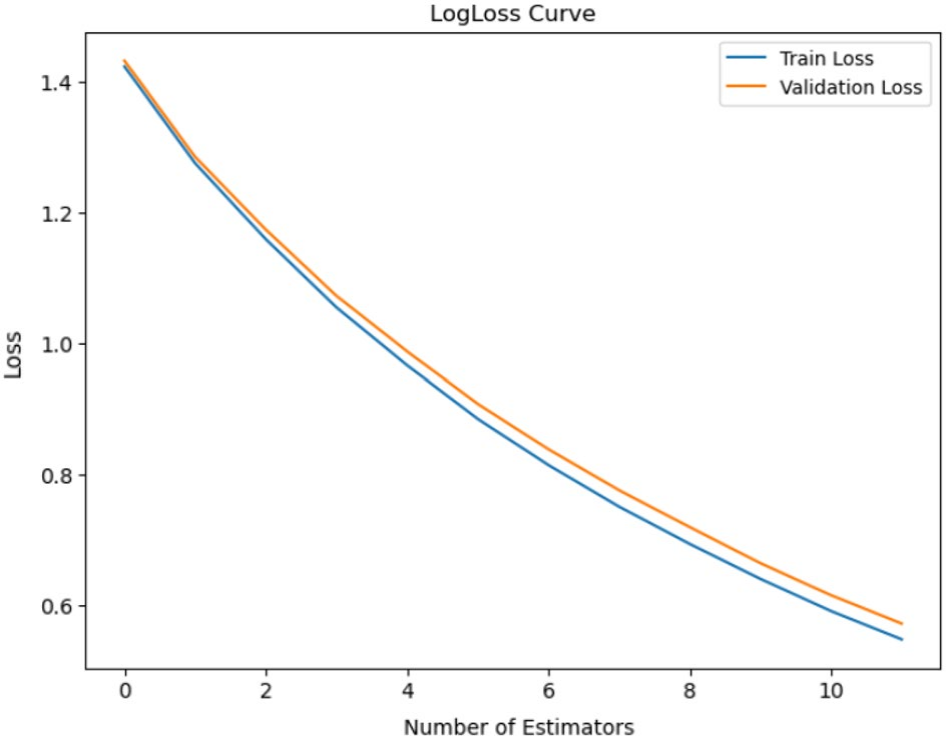
The classification model’s log loss representation shows the logarithmic loss metric over epochs or iterations, giving information about the model’s convergence and predictive uncertainty during the training and validation stages.

Regarding our pursuit of VF categorization, our reported loss is currently negligible at 0.4, where the validation loss is recorded as 0.5. Loss is a quantifiable measure of the degree to which a model’s predictions match actual values in the training data. It measures the discrepancy between forecasted and actual results. Training aims to reduce this loss, and improve the capacity of the model to generate precise predictions. Figure 11 illustrates these training and validation loss with number of epochs.

#### Error rate curve of classification model

**Figure 12:**
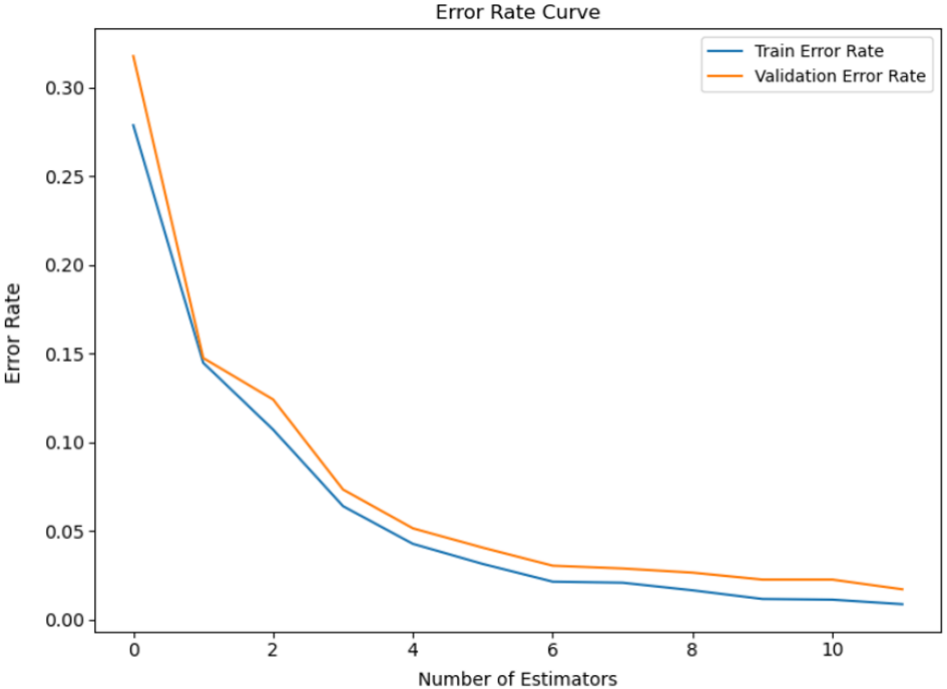
The classification model’s error rate curve illustrates the learning trajectory and convergence of the model by showing the evolution of the model’s performance over training iterations or epochs.

The error rate curve (ERC), a learning curve, is essential in machine learning to evaluate model performance during training. The graph illustrates the correlation between the model’s training and validation error rates for several epochs or iterations. Tracking the error rate curve helps detect problems such as overfitting and underfitting, assists in hyperparameter optimization, and pinpoints the optimal threshold where the model performs well on new, unseen data. Regarding our endeavor to classify VFs, the ERC demonstrates encouraging outcomes: a training error rate of 0.2 and a validation error rate of 0.3. Figure 12 shows the error rates in a graph as part of our investigation.

#### Performance metrics measurement for classification model

Precision, recall, and F1 scores are crucial metrics employed to evaluate the efficacy of machine learning models. Precision assesses the accuracy of optimistic forecasts by ensuring that the identified positives are positive. Recall quantifies the ability of a model to correctly identify all relevant occurrences, explicitly measuring the proportion of real positive cases that are correctly predicted. The F1 score balances precision and recall by calculating their harmonic mean. These measures are essential for understanding the efficiency of a model, especially in activities where the precise identification of positive instances is extremely important, such as medical diagnosis or fraud detection. They contribute to creating models that achieve an ideal balance between false positives and false negatives. Thereby, improving the overall effectiveness and reliability. The computing formula as follows:

26 $$\begin{aligned} Precision= & {} \,\frac{TP}{TP+FP} \end{aligned}$$

27 $$\begin{aligned} Recall= & {} \,\frac{TP}{TP+FN} \end{aligned}$$

28 $$\begin{aligned} F1\,score= & {} \,2*\frac{Precision*Recall}{Precision+Recall} \end{aligned}$$

Table 5 displaying testing precision, recall, and F1 score provides a clear visual representation of a machine learning or classification model’s performance. Here, precision can be slightly lower than recall in this case due to the model’s tendency to be more conservative in its positive predictions. It prioritizes accuracy in positive predictions (precision) over capturing all positive instances (recall).

**Table 5:** The model’s performance metrics, which exhibit precision, recall, and F1-score measurements for each class in the VF disease classification, demonstrate how well the efficientNetV2L-LGBM classifier distinguishes between various VF diseases.

Class	Precision	Recall	F1-score
Carcinoma	0.95	0.98	0.97
Dysphonia	0.99	0.96	0.97
Healthy	0.98	0.97	0.98
Paresis	0.97	0.98	0.97
Polyp	1.00	1.00	1.00

#### Confusion matrix of classification model

**Figure 13:**
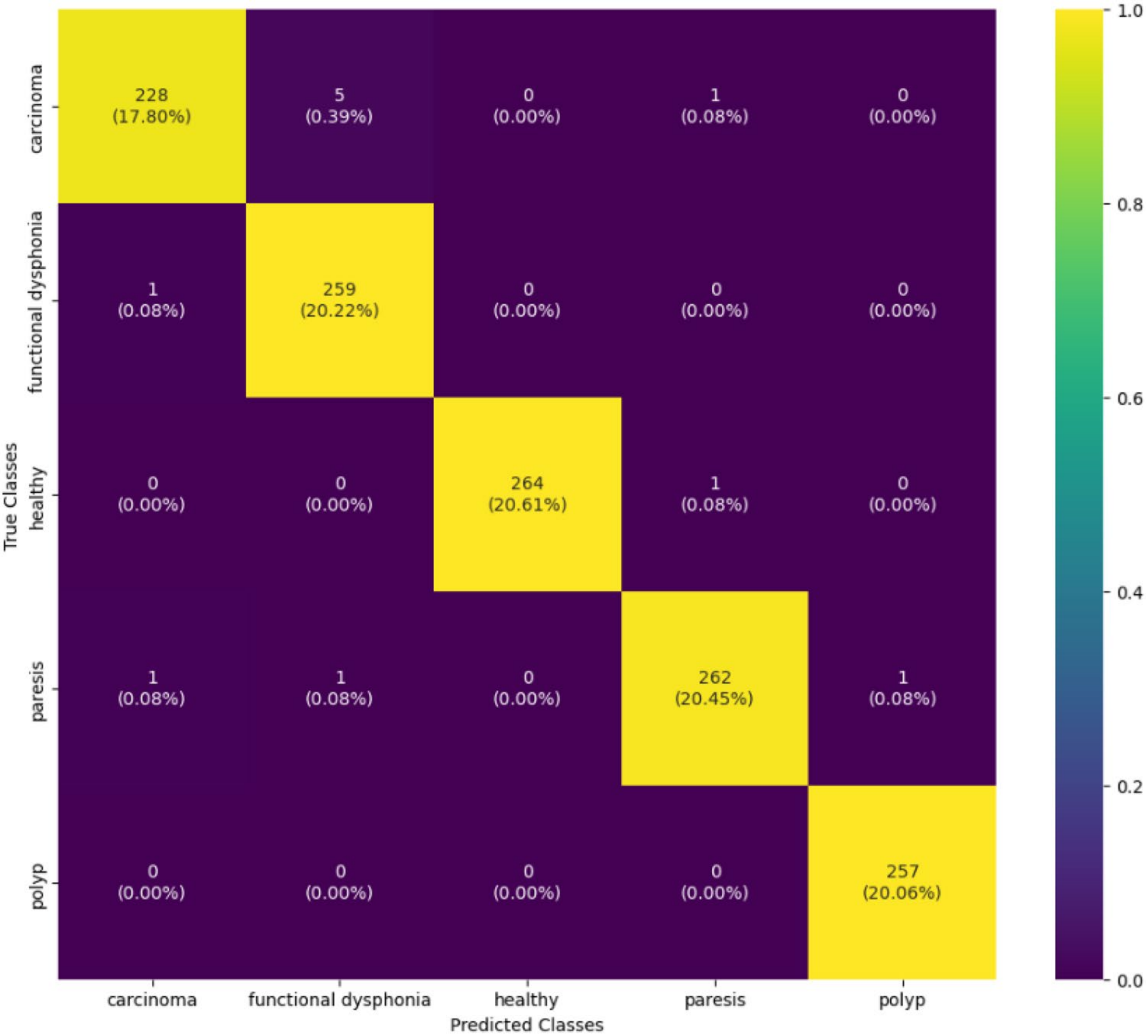
The confusion matrix of the classification model offers valuable insights into the model’s strengths and shortcomings in categorizing various categories. It illustrates the performance evaluation by showing the distribution of predicted versus actual class labels.

An essential machine learning tool for assessing the efficiency of a classification model is a confusion matrix. The confusion matrix displays the actual and predicted rates for the five classes: carcinoma, dysphonia, paresis, polyp, and healthy, offering a thorough evaluation of the model’s performance. This assessment helps to measure the precision of forecasts in many categories, improving the comprehension of the model’s efficiency in differentiating between carcinoma, dysphonia, paresis, and polyp cases. The matrix is generally structured in a tabular arrangement with rows and columns denoting the categorized and actual classes. The yellow box symbolizes the class most likely to generate misunderstanding, whereas the purple box represents the class that is least likely to produce confusion. Figure 13 shows the precision of the categorization model.

#### Performance measure of implemented models examined for classification model

Our ensemble EfficientNetV2L-LGBM model is distinguished by its exceptional accuracy among many machine learning models for classifying VF disease. The performance of this classification model indicates its efficiency and accuracy. We have effectively applied various sophisticated models to our categorization challenge, all of which have produced impressive outcomes. Our ensemble model, which combines EfficientNetV2L with LGBM, has proven to be a top performer. The training accuracy was 98.88%, and the validation accuracy was 97.73%, demonstrating the strength of the proposed approach. Its outstanding test accuracy of 97.88% demonstrates the efficiency of the classification. This significant accomplishment highlights the accuracy and dependability of our categorization approach, particularly in the field of vocal illness diagnosis. Table 6 presents the results of the implemented models.

**Table 6:** Comparative analysis of validation, test, and training accuracy across various implemented classifier models, which illustrates the generalization and performance differences among the models created for our classifier and helps with robustness assessment and model selection.

Model/classifier	Training accuracy (%)	Validaion accuracy (%)	Test accuracy (%)
EfficientNetV2L and XGBoost	95.68	87.39	90.43
EfficientNetV2L and SVM	96.94	93.65	93.91
ResNet50 and XGBoost	81.97	72.39	71.87
ResNet50 and LGBM	95.81	94.72	95.43
ResNet50 and SVM	87.26	82.59	81.79
ResNet50	91.33	87.48	89.12
VGG16	68.75	65.61	65.72
VGG16 and XGBoost	58.82	49.95	51.93
VIT and XGBoost	83.45	79.16	77.77
VIT and LGBM	81.96	79.65	81.66
EfficientNetV2L and LGBM (proposed)	**98.88**	**97.73**	**97.88**

Significant values are in bold.

### 10-Fold cross validation implementation for classification task

We employ a tenfold cross-validation technique to evaluate the performance of the classification model. 10-fold cross-validation is essential for classification problems to guarantee a reliable model performance assessment. The data were partitioned into ten subgroups, and the model was trained on nine subsets while repetitively validating the remaining subset. This procedure is iterated with distinct subgroups to mitigate variability and diminish the likelihood of overfitting or underfitting. It offers a more dependable evaluation of model performance and aids in efficiently adjusting the hyperparameters, resulting in a more transferable model. During the ninefold cross-validation, we recorded a validation accuracy 98.85%, demonstrating the model’s exceptional performance on previously unexplored data. In addition, tenfold cross-validation resulted in a training accuracy of 99.75%, indicating that the model performed exceptionally well on the training dataset. Table 7 displays the detailed outcomes of tenfold cross-validation, emphasizing the accuracy measurements for each fold of the dataset. These findings highlight the model’s competence in learning from data while maintaining a strong generalization ability.

**Table 7:** Result of our proposed model EfficientNetV2L-LGBM in image classification using k-fold cross validation.

Fold	Training acuracy (%)	Validation acuracy (%)
1	74.82	77.62
2	86.96	84.79
3	89.21	90.56
4	92.45	92.69
5	95.65	96.08
6	96.89	96.93
7	96.65	97.78
8	98.19	97.45
9	99.55	** 98.85**
10	**99.75**	97.98

Significant values are in bold.

#### Comparison of existing and proposed model results for classification task

After a thorough examination, we conducted a complete comparison of different well-established models that were specifically developed to identify VF disease. Our primary goal in the VF categorization competition was to attain precise and accurate outcomes. We comprehensively analyzed many cutting-edge models specifically developed for the classification of VF disease, drawing upon a vast body of pertinent literature. Each of these models offers distinct and diverse techniques for classifying VF diseases, using a range of architectural designs and scientific methods. We suggest performing a comparative analysis to evaluate the effectiveness and outcomes of our innovative ensemble EfficientNetV2L-LGBM model compared with other existing approaches for diagnosing VF conditions. This comprehensive study allowed us to assess the strengths and weaknesses of our model compared to the current ones, ultimately highlighting its appropriateness for addressing the intricate issue of VF disease classification. Table 8 presents a comprehensive overview of the several established techniques used to classify vocal folds, along with the corresponding model and test accuracy of each proposed approach.

**Table 8:** Comparing the performance of current and suggested models for vocal fold disease classification.

Reference	Model	Test accuracy (%)
^40^	SVM	82.14
^41^	DCNN	80.23
^42^	ANN	83.58
^43^	ResNet	90
^44^	SVM	94.28
^45^	SVM	92
(Proposed)	**EfficientNetV2L-LGBM**	** 97.88**

Significant values are in bold.

### Results of ensemble UNet-BiGRU as segmentation model

In this section, we show the segmentation results of the UNet-BiGRU model. Key performance parameters are shown, such as the training loss, validation loss, training accuracy, and validation accuracy. We also emphasize the usefulness of accuracy, which is an essential metric for assessing the segmentation criteria.

#### Accuracy and loss of vocal fold segmentation

**Figure 14:**
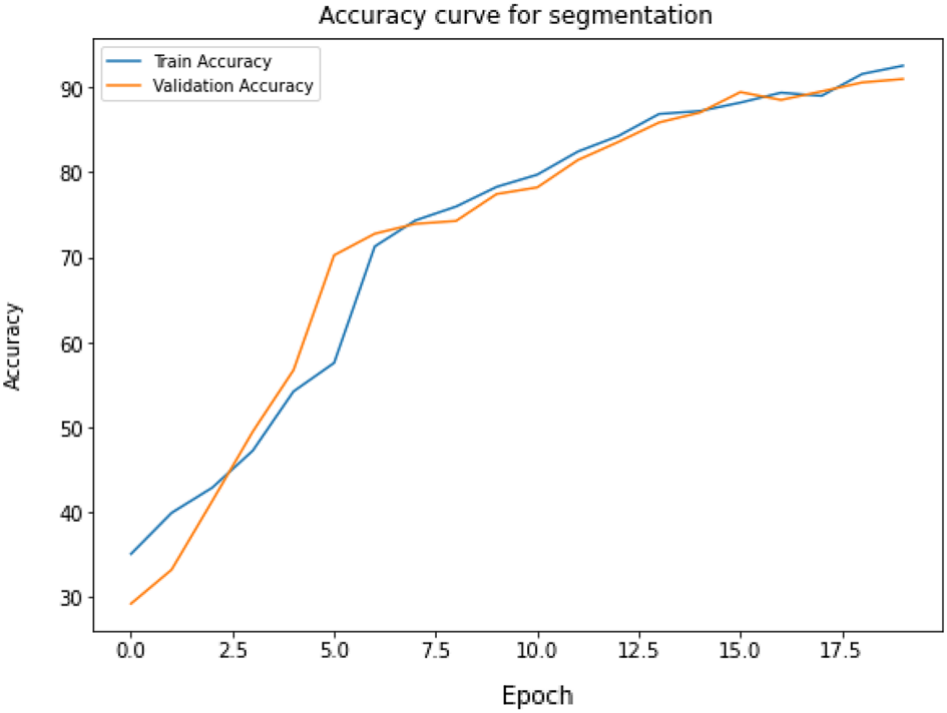
VF segmentation using UNet-BiGRU: training and validation accuracy curve, which shows the learning process and generalization ability of the segmentation model by showcasing the performance trends and comparative accuracy between training and validation sets throughout the model’s training epochs.

We have achieved a remarkable training accuracy of 92.55% in our efforts to segment VF. Additionally, our validation accuracy was 89.87% and a practical test accuracy of 91.47%. The results successfully demonstrated the proficiency of the segmentation training and validated its performance. Their analysis confirms proficiency of the segmentation model in appropriately categorizing segmented items during the training and validation phases. Figure 14 presents the traning and validation accuracy of segmentation model.

**Figure 15:**
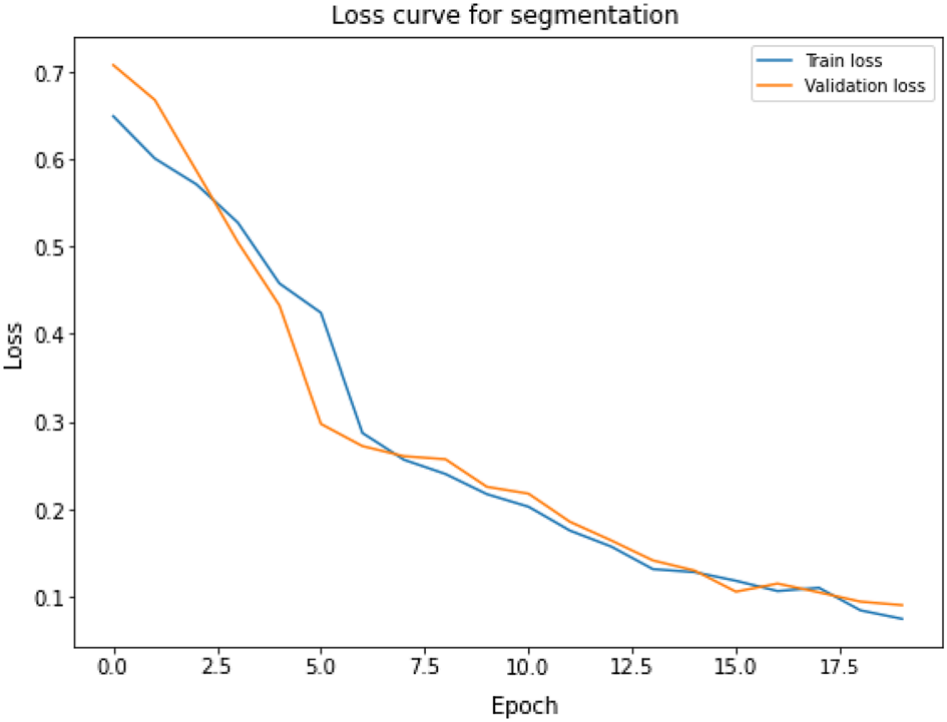
Training and validation loss curve for the segmentation model: this figure illustrates the loss trends for each training epoch for the training and validation datasets, showing how the segmentation model is convergent and learning.

Regarding our pursuit of VF segmentation, our reported loss is currently at a negligible 0.01, while the validation loss is recorded as 0.02. The quantitative indicator of how well a model matches the actual values in the training data with its predictions is called the loss. It measures the discrepancy between actual results and predictions. To improve the predictive accuracy of the model, the training process aims to minimize this loss. Figure 15 illustrates the training and validation loss of segmentation model.

#### Precision curve of segmentation model

**Figure 16:**
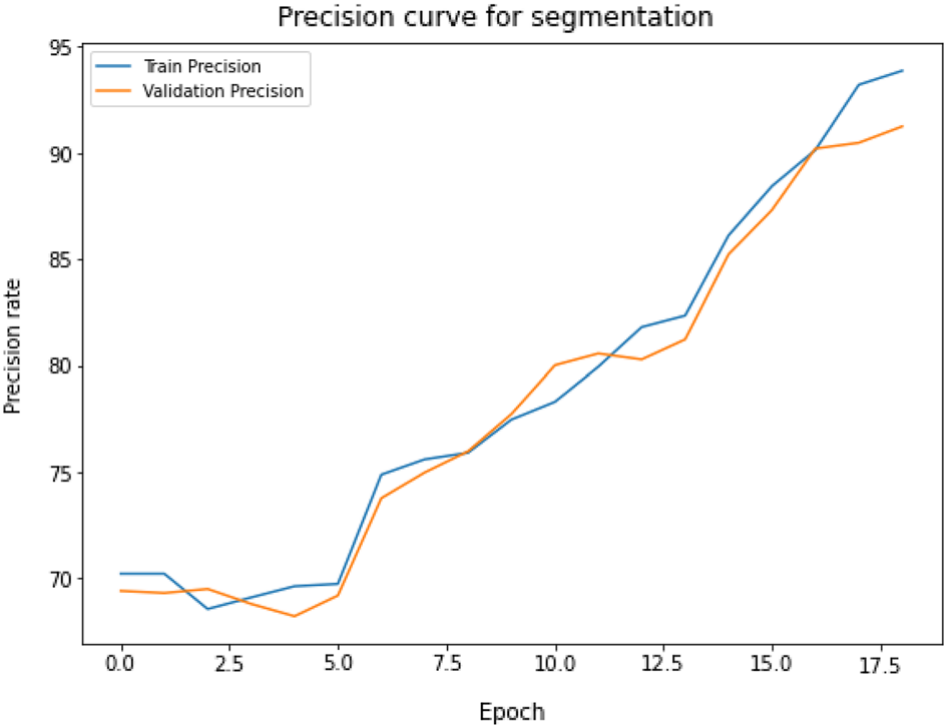
The precision levels attained during the segmentation process are revealed by the training and validation precision curve for vocal fold segmentation using UNet-BiGRU, which shows the precision trends and comparative performance between training and validation datasets across model training epochs.

The segmentation procedure demonstrates outstanding precision, highlighting the efficiency of our approach. The performance of the proposed model is exceptional, with a training precision of 94.67% and a validation precision of 91.63%. The high precision scores highlight the solid and consistent performance of our segmentation model, confirming its dependability and precision. Figure 16 clearly illustrates the depiction of these precision measurements, highlighting the effectiveness and excellence the proposed of our segmentation technique. The segmentation procedure demonstrates outstanding precision, highlighting the efficiency of our approach. The performance of the proposed model is exceptional, with a training precision of 94.67% and a validation precision of 91.63%. The high precision scores highlight the solid and consistent performance of our segmentation model, confirming its dependability and precision. Fig. 16 clearly illustrates the depiction of these precision measurements, highlighting the effectiveness and excellence of proposed segmentation technique.

#### Performance measure of implemented models for segmentation task testing

We have rigorously utilized several methodologies to evaluate the precision of VF segmentation. The results indicate a positive outcome: our test accuracy is 91.47%. Furthermore, our Testing precision, a vital measurement, stands out at an impressive 91.99%, indicating our segmentation process’s exceptional quality. Upon further examination, the IOU statistic demonstrates a significant 87.46% value, highlighting the effectiveness of our method in precisely outlining the VF. The sensitivity, a crucial metric, exhibits an admirable accuracy rate of 85.76%, while the specificity achieves a remarkable value of 79.98%, indicating a comprehensive ability to differentiate genuine negatives. In addition, the dice score, which measures the similarity of segmentation, is recorded at a significant 71.52%. Combining an exceptional F1 score of 88.76% further strengthens our segmentation model’s credibility and robustness. The results confirm the efficacy of utilizing a fusion model such as UNet and BiGRU, emphasizing their contribution to obtaining precise and efficient VF segmentation. Table 9 presents a comprehensive analysis of the performance measures based on several characteristics, further emphasizing our segmentation process’s innovative and accurate quality. This thorough assessment establishes our methodology as a significant breakthrough in the area, establishing a higher benchmark for exceptional segmentation.

**Table 9:** A comparative examination of segmentation model testing results includes metrics such as test accuracy, precision, IOU, sensitivity, specificity, dice coefficient, and F1 score.

Model	Accuracy (%)	Precision (%)	IOU (%)	Sensitivity (%)	Specificity (%)	Dice (%)	F1 score (%)
UNet	86.46	70.23	81.87	76.03	95.44	58.16	73.01
UNet and LSTM	87.07	72.21	83.35	79.33	91.56	57.88	75.60
DeepLabv3+ and ResNet50	89.81	89.52	65.93	80.35	92.41	62.86	84.68
DeepLabv3+	87.35	76.59	80.21	76.77	91.52	65.22	76.67
UNet and MobileNet	78.45	82.37	61.29	76.43	96.92	33.52	79.28
ResUnet	88.46	69.42	56.92	76.85	65.78	47.03	72.94
UNet and ResNet50	88.85	75.18	82.39	81.69	86.91	65.67	78.29
UNet and ResNet101	90.89	86.85	77.63	82.95	82.82	63.69	84.85
UNet-BiGRU (proposed)	**91.47**	**91.99**	**87.46**	**85.76**	**79.98**	**71.52**	**88.76**

This document presents the outcome of the segmentation task on the test set.

Significant values are in bold.

### 10-fold cross-validation implementation for segmentation task

Employing tenfold cross-validation for our UNet-BiGRU model was essential to thoroughly assess its segmentation performance while efficiently mitigating potential overfitting concerns. During the experiment, each fold was used as a separate validation set to thoroughly evaluate the performance of the model on different subsets of the data. Significantly, in fold 8, we observed promising outcomes with a training accuracy of 96.95% and a validation accuracy of 92.56%. Although these metrics demonstrate the model’s capacity to learn from the training data, a minor decrease in the validation accuracy relative to training accuracy indicates the potential occurrence of overfitting. Table 10 showcases the tenfold cross-validation experiment for the segmentation model and the performance indicators for each fold. This methodology guarantee the strength and adaptability of the model by methodically verifying its efficacy with different subsets of data.

**Table 10:** Accuracy of our proposed model UNet-BiGRU in image segmentation using k-fold cross validation.

Fold	Training accuracy (%)	Validation accuracy (%)
1	35.28	32.74
2	49.65	38.54
3	56.22	48.67
4	68.57	59.69
5	79.68	72.77
6	88.47	85.33
7	92.78	87.46
8	**96.95**	**92.56**
9	95.56	90.65
10	95.84	90.89

Significant values are in bold.

#### Segmentation results representation on true mask and predicted mask

**Figure 17:**
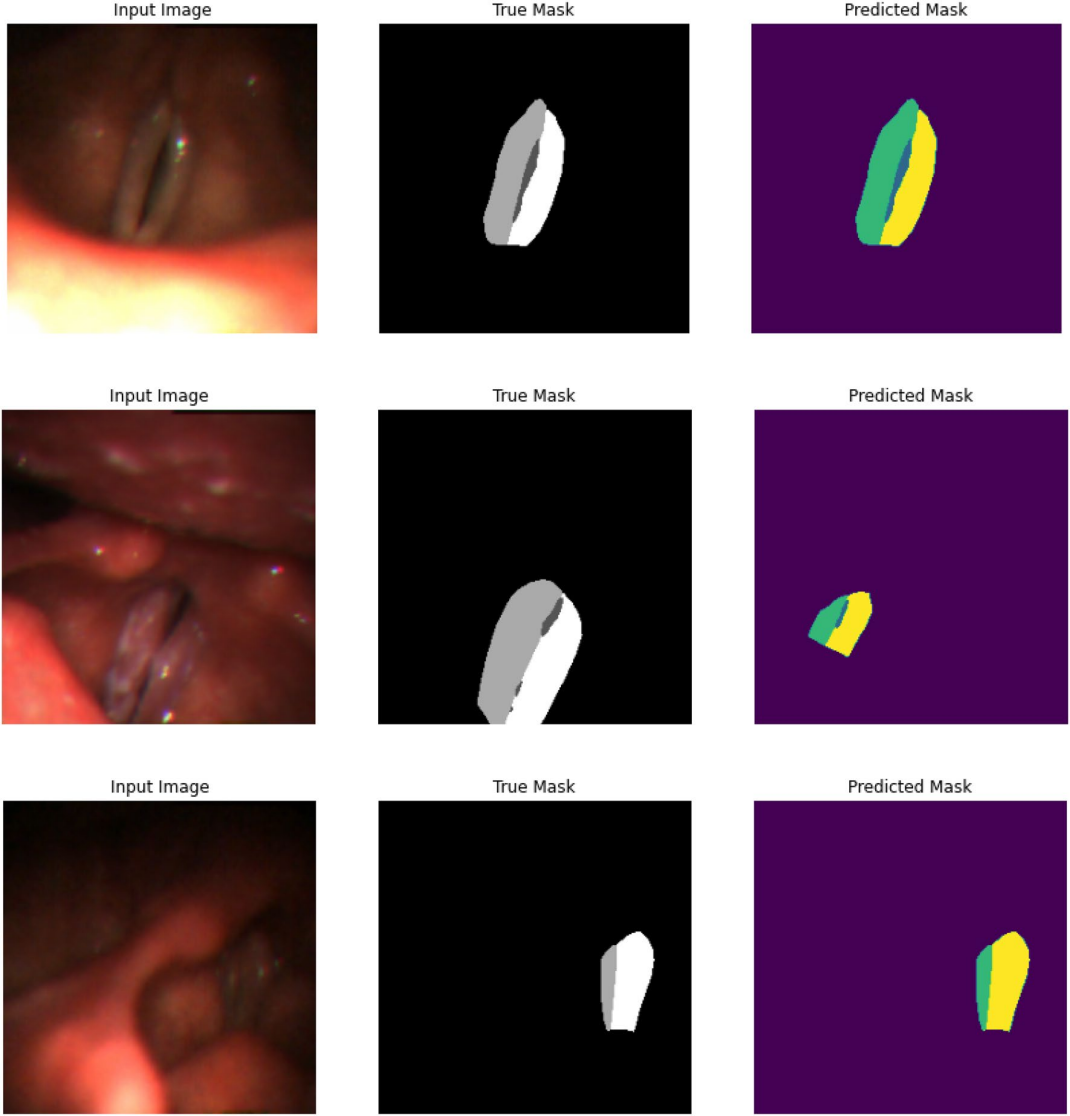
The input image, matching ground truth mask, and predicted mask are displayed, illustrating the model’s ability to correctly segment vocal folds from medical imaging data. VF segmentation results are visualized using UNet-BiGRU.

Our segmentation technique includes the production of true and predicted masks based on the original VF data. These masks demonstrate the efficacy of the proposed segmentation approach. The segmentation process involves breaking an image into meaningful sections, and assessing the accuracy of the segmentation models requires knowledge of the actual and predicted masks. The model produces the predicted mask, whereas the genuine mask serves as the ground truth by accurately segmenting the image. By comparing these masks, one can evaluate the model’s performance and make improvements to segmentation algorithms by determining how well it recognizes and distinguishes objects or regions inside the image. In this section, we present the segmentation results of the UNet-BiGRU model. Key performance parameters are shown, such as the training loss, validation loss, training accuracy, and validation accuracy. We also emphasize the usefulness of accuracy, which is an essential metric for assessing segmentation criteria. We demonstrated the precision and effectiveness of our segmentation process by combining the original images with their true and predicted masks. This comparison confirms the excellence of our segmentation and demonstrates its capacity to accurately define and recognize complex structures within the VF data. Figure 17 clearly illustrates this approach, displaying input images with their respective true and predicted masks. There are two sides in the vocal fold area, the left and right vocal folds, with the middle space being the trachea. In the true mask, dark grey represents the trachea. Additionally, the light grey region indicates the affected side of the vocal fold, while the white region represents the healthy side. Similarly, in the predicted section, the green and yellow regions correspond to the predicted affected areas, and predicted affected areas, respectively.

### Comparison of existing and proposed model results for segmentation task

When comparing our segmentation work in vocal fold analysis, our UNet-BiGRU model emerges as a leading contender, showcasing exceptional performance. Our model demonstrated its effectiveness in reliably identifying vocal fold structures, with an impressive testing accuracy of 91.47%. The testing precision is an astonishing 91.99%, further highlighting the model’s capability to identify and categorize key characteristics accurately. In addition, the IOU score, which is a crucial measure in segmentation tasks, achieves an impressive 87.46%. These significant accomplishments demonstrate the efficacy of our segmentation method, establishing our UNet-BiGRU model as a reliable solution for vocal fold segmentation tasks. Table 11 presents a comparison of the segmentation studies.

**Table 11:** Comparing the performance of current and suggested models for vocal fold segmentation.

Reference	Model	Improvement
^46^	CNN-LSTM	Dice 0.85, Glottis 0.91%
^28^	ML	Sensitivity of 86%, 94%, 80%, 73%, and 76%
^42^	ANN	Test accuracy 83.58%
^47^	DL	Sensitivity 0.85, specificity 0.85
^48^	SVM	Dice 92.9%, sensitivity 93.5%, precision 92.6%
^49^	FTriangNB	Test accuracy 87.5%
^50^	UNet and ErfNet	IoU 84.7%
(Proposed)	**UNet-BiGRU**	** Accuracy 91.47%, Precision 91.99%, IOU 87.46%**

Significant values are in bold.

## Discussion

Regarding the field of healthcare, categorization of VF illnesses is a crucial matter of concern. The segment of the vocal fold is crucial and considered essential in this undertaking. We have undertaken this dual endeavor within our system with a clear and determined objective. We aimed to categorize five specific conditions affecting VF: carcinoma, dysphonia, paresis, polyp, and standard VF categorization. Our method demonstrates exceptional proficiency in identifying and segmenting diverse VF structures. By converting the image data to grayscale, we simplified the process of extracting essential features, enabling our model to concentrate on significant visual elements while reducing the impact of color differences. To enhance the robustness of our model against overfitting, we implemented a standardization approach. This process convert the signal from each image channel into a stochastic variable with an average value of 0 and a standard deviation of 1. Rigorous standardization resolved problems related to overfitting and contributed to achieving data balance, resulting in a strong and resilient model. By utilizing the effectiveness of an Ensemble model, we combined the effectiveness of EfficientNetV2L-LGBM for classification and utilized the capabilities of an ensemble UNet-BiGRU for VF segmentation. Analyzing the computational time of our system uncovers intriguing observations. Our model efficiently handled an extensive dataset of 12,810 photos in just 129.5 seconds, demonstrating its capability in the classification challenge. The segmentation of 24,000 photos was completed in 2726.3 seconds. The extend time emphasizes the computational requirements linked to better datasets and intricate image processing operations. These findings emphasize the significance of enhancing the performance of our system significantly when expanding to accommodate larger datasets and more complex tasks such as image segmentation. To improve the overall efficiency and scalability of our model for demanding tasks in the future, it is essential to allocate resources efficiently and make algorithmic improvements. We present our segmentation and classification results, demonstrating outstanding performance for multiple criteria. Our technology demonstrates its efficacy and reliability by effectively classifying and precisely segmenting vocal folds. These findings highlight the high-level performance of our system in segmentation and classification tasks, particularly for vocal fold analysis. Machine learning has the potential to significantly transform the classification and division of vocal fold problems in the medical domain. Machine learning algorithms can be trained to effectively categorize various illnesses by utilizing extensive datasets of vocal fold photos, videos, and patient records and analyzing minor visual and auditory clues. These algorithms can surpass previous manual procedures by offering more accurate and consistent diagnostic capabilities. Moreover, machine learning can expedite the identification of vocal fold anomalies, resulting in prompt interventions and enhanced patient outcomes. Automating this procedure can also relieve the workload of healthcare personnel, enabling them to devote more attention to patient care and treatment planning. However, the use of machine learning technology in this field holds the potential to improve the precision, effectiveness, and availability of diagnosing and treating vocal fold problems. Combining classification and segmentation results is crucial in improving diagnostic accuracy and healthcare for vocal fold disorders. Clinicians can better understand the specific nature of a patient’s condition by accurately classifying different types of vocal fold pathologies based on imaging or acoustic data. Segmentation further refines this understanding by precisely delineating the affected areas within vocal folds. This detailed localization helps assess the extent and severity of the disorder. Together, classification and segmentation outcomes enable more tailored treatment plans, and guide interventions such as surgery or therapy with greater precision. A advancements in diagnostic accuracy driven by these technologies lead to earlier detection and more personalized patient management strategies. This improves patient outcomes by reducing misdiagnosis and unnecessary procedures and enhancing overall healthcare efficiency in the field of vocal fold disorders. Several challenges arose as we were putting our system into operation. First of all, there were many obstacles at the data preprocessing stage. We used advanced methods to efficiently preprocess our data. Second, the experiments we did required significantly more rigorous hyperparameter tuning. The optimal parameters for enhancing the performance of our model require significant trial and error. We successfully determined which parameter values were optimal and efficient. Third, customizing the proposed model was a challenging task. To develop an ensemble model, we added extra layers and modified them to meet our needs. Creating an innovative, fully customized model was a challenging task. Finally, a major challenge was obtaining our model to generate consistent results. Despite the intricacy, we persevered and could accurately ascertain the outcomes for our system. Ultimately, these challenges have contributed to the development of robust and workable solutions.

## Conclusion and future work

Our study distinguishes itself by developing an automated system designed explicitly for VF disease classification and VF segmentation. We have successfully classified disorders such as carcinoma, dysphonia, paresis, polyps, and healthy VFs. Our segmentation technique effectively and precisely identifies complex VF structures. We implemented an approach that utilized an ensemble EfficientNetV2L-LGBM model for VF disease classification and an ensemble UNet-BiGRU model for VF segmentation. We attained significant accuracy in the categorization process, guaranteeing meticulous identification of diseases. Furthermore, our segmentation approach achieved exceptional accuracy, precision, and IOU, thereby introducing new and precise limits for VF structures. The deployment of this VF segmentation and categorization method represents a substantial advancement in the medical healthcare technology.

In the future, more datasets will be employed to automate this domain further. Additionally, video data will be utilized to put the system into operation. More sophisticated models will be developed within this system to identify patients with VF disorders accurately.

## Data Availability

Data is available in a publicly accessible link: https://zenodo.org/records/3603185.
